# The Ins and the Outs

**Published:** 1882-12-20

**Authors:** 


### *THE INS AND THE OUTS.*

In medicine as in politics, the Outs are always assailing the Ins, hence we hear continued complaint in Medical Journals against Medical Colleges in regard to bad management—imperfect teaching and the laxity of requirement in conferring diplomas. It is freely conceded that a regular standard of education is desirable, and that most colleges are not sufficiently rigid and careful in conferring degrees; yet it must be borne in mind that the fault is not so much with the colleges as with the profession and the people.

Our systems of education, whether in medicine or other departments, are all imperfect, undeveloped and faulty. The country is new. Most of our young men in the South desiring professions are poor, and the State now lends no aid or encouragement to the improvement of medical education. On the contrary there are laws obstructing anatomical investigation. While this is all true, yet it is also true that the average Doctor in America, yea, even in the Southern States, is not a whit behind that of other countries. Medical discoveries are as frequent, and medical advance as rapid in this country as in any other portion of the globe, and the writer of this article, a graduate of an old and reputable Northern institution, will doubtless be sustained in the assertion by every physician, even the most eminent among us—who graduated at the best Northern schools—that here in Atlanta as good facilities, nay, better facilities can be found for procuring a medical education than were furnished in their day by the older and more renowned Northern

Institutions. And yet, many of those who graduated North under these meagre advantages, are successful and emminent men in the profession. After all, it is not so much in the school as in the man. If the student possesses the qualities of head and heart, and the spirit of study and energy which is essential to success, he will, in due time, achieve it. If he does not he will fail, and go out of the profession as thousands do, and as they ought to do.

